# Exposure to pesticides during development negatively affects honey bee (*Apis mellifera*) drone sperm viability

**DOI:** 10.1371/journal.pone.0208630

**Published:** 2018-12-13

**Authors:** Adrian Fisher, Juliana Rangel

**Affiliations:** Department of Entomology, Texas A&M University, College Station, Texas, United States of America; Institut Sophia Agrobiotech, FRANCE

## Abstract

Honey bee (*Apis mellifera*) colonies invest a substantial amount of colony resources in the production of drones during the reproductive season to enable mating with virgin queens from nearby colonies. Recent studies have shown significant differences in the production of sperm cells that are viable (i.e., sperm viability) and can fertilize an ovule among sexually mature drones that are exposed to different environmental conditions during development or as adults. In particular, sperm viability may be negatively affected during drone development from exposure to pesticides in contaminated beeswax. To assess whether sperm viability is negatively affected during drone development from exposure to beeswax contaminated with in-hive pesticides, we compared the viability of sperm collected from drones reared in pesticide-free beeswax with that of drones reared in beeswax contaminated with field-relevant concentrations of the pesticides most commonly found in wax from commercial beekeeping operations in the United States. These pesticides include the miticides fluvalinate, coumaphos and amitraz, and the agro-chemicals chlorothalonil and chlorpyrifos. Sperm from drones collected at 10 and 18 days post emergence were classified as viable or non-viable to calculate sperm viability. For all pesticide treatment groups, drones that were reared in pesticide-laden beeswax had lower sperm viability compared to those reared in pesticide-free beeswax. This difference was especially pronounced among drones reared in miticide-laden wax. Our results reinforce the notion that pesticide contamination of beeswax negatively affects the reproductive quality of drones, which can affect the queens they mate with, ultimately compromising colony health.

## Introduction

Among species of eusocial Hymenoptera (ants, wasps, and bees), males live sheltered lives inside their hives, where they are raised by sister workers until they are ready to mate [1]. A substantial amount of colony resources is invested in the care and nurturing of males, despite the fact that they provide no contributions to colony maintenance apart from reproduction [2]. Male rearing by workers appears regularly among eusocial insects [3], especially in swarm-founding species such as honey bees in the genus *Apis*, which exhibit an extreme male-biased sex ratio in reproductive individuals [4,5]. In the honey bee, *Apis mellifera*, colonies are composed of one queen and a few thousand seasonal males (drones), which are only reared during the reproductive season when colony resources are plentiful [4]. Virgin honey bee queens mate with an average of 12 to 15 drones [6], collecting and depositing 4 to 7 million sperm cells for up to five years in a sperm-storing organ known as the spermatheca [7–9]. During the reproductive season thousands of drones congregate up in the air where they locate and attempt to mate with virgin queens from nearby, unrelated colonies [4]. As competition among drones for a chance to mate is intense, those individuals with higher sexual competitiveness are likely to have higher reproductive fitness compared to less competitive ones [5]. For example, drone weight and size have been found to significantly correlate with greater mating success and paternal representation in a queen’s progeny [10]. But, despite the importance of drones as reservoirs of a colony’s genetic traits, few studies have looked at the effects of environmental factors on drone reproductive quality.

Exposure to pesticides is one of the environmental factors that could negatively impact drone sperm viability. Studies examining fipronil, a systemic insecticide, discovered significant repercussions to drone reproductive health following exposure, including a reduction in sperm viability [11,12,13]. Additionally, exposure to fipronil reduced sperm production while conversely increasing sperm metabolism [11]. Fipronil also conferred synergistic effects on drones following co-exposure with the microsporidian parasite *Nosema ceranae*, increasing negative impacts on neural, metabolic, and detoxification processes in tagma and the midgut [12]. Furthermore, the neonicotinoid insecticide imidacloprid negatively impacted hemolymph antioxidant capacity in drones at different ages [14]. However, the seminal plasma exhibited a lower antioxidant capacity than hemolymph and thus, seminal plama be more sensitive to the stresses of insecticide exposure [14].

In a study examining 250 commercial beekeeping operations in the United States, Mullin et al. (2010) [15] found dozens of pesticides in beeswax samples, some at high concentrations. The group of chemicals used to combat the honey bee ectoparasitic mite *Varroa destructor* is especially troubling because they were the most prevalent pesticides found in those samples. When *Varroa* mites are found in large numbers and left untreated, colonies collapse and often die [16]. Continuous treatment of colonies against *Varroa* mite infestations has led to most mite populations becoming resistant to the miticides most commonly used in the last two decades [17,18], and has caused escalating and prolonged contamination of the wax inside hives [15]. But despite their ubiquitous presence in wax, few studies have explored the effects of exposure to these pesticides on drone reproductive quality. Exposure to the pyrethroid fluvalinate and the formamidine amitraz, which are active ingredients of many *Varroa* control products used in the beekeeping industry, has been found to lower drone body weight and mating flight frequency [19,20]. In addition, fluvalinate and amitraz, as well as the organophosphate coumaphos, cause lower drone sperm counts and viability [20,21].

Interestingly, Johnson et al. (2013) reported no impact of six miticides (fluvalinate, coumaphos, fenpyroximate, amitraz, thymol and oxalic acid) on drone sperm viability [22]. However, in that study, miticides were applied topically on one- to four-day-old adult drones, thus leaving a knowledge gap on the potential effects of exposure to miticides during development on drone reproductive quality. It is particularly important to fill this gap because new adult drones emerge from their cells with all the sperm they will ever possess [5,23]. In fact, they undergo only minor anatomical changes after emergence [24], an aspect of male biology found in other hymenopteran species [2]. Therefore, we hypothesize that environmental conditions faced by drones during development may affect their reproductive fitness as adults. Incidentally, because the comb within hives is typically contaminated with several miticides at once [15], studies on the effects of pesticide contamination of the wax environment on drone reproductive quality should build on the aforementioned findings and focus on combinations of these chemicals, not simply focus on one product at a time.

Several agro-chemicals have also been found to negatively impact drone reproductive health. In particular, oral exposure to the neonicotinoid insecticides thiamethoxam and clothianidin are known to decrease sperm viability in adult drones [25]. Similarly, exposure to imidacloprid was found to affect drone sperm viability and mitochondrial activity, although the intensity of the effect also varied significantly between colonies [26]. Together, these findings show that exposure to field-applied pesticides, either through contamination of the beeswax substrate used for brood rearing, or through consumption of contaminated food, negatively affects drone sperm counts and viability.

In this study, we reared drones using frames of plastic foundation coated with wax that was either pesticide-free or contaminated with field-relevant concentrations of the five most ubiquitous agrochemicals found in wax samples (i.e., the miticides amitraz, coumaphos and fluvalinate, and the pesticides chlorpyrifos and chlorothalonil) collected from over 250 commercial beekeeping operations across the United States [15]. We then measured sperm viability in adult drones that were reared in either pesticide-free or pesticide-laden wax. We make recommendations regarding the use of these pesticides near or within honey bee colonies based on our findings.

## Materials and methods

This study was conducted during the 2016 and 2017 reproductive seasons at the Janice and John G. Thomas Honey Bee Facility of the Texas A&M University RELLIS Campus in Bryan, TX. To stimulate the production of experimental drones, we introduced plastic drone foundation frames into strong colonies to encourage drone rearing. The frames were previously coated with melted beeswax following the procedures outlined below.

### Drone frame preparation

To prepare the frames used for drone rearing, approximately 20 lbs of organic, cosmetic grade beeswax pellets (Koster Keunen Inc., Watertown, CT, USA) were melted in a large water bath. Once the wax was melted, plastic drone foundation frames (Brushy Mountain Bee Farm, Moravian Falls, NC) were submerged individually into the molten wax once or twice until they were fully coated. Each frame was then allocated to an experimental group.

In 2016, eight drone foundation frames were allocated to the amitraz treatment group and eight to the untreated control group. Frames in the control group were sprayed with 10 mL of acetone on both sides using a 750 mL all-purpose sprayer. Each frame allocated to the amitraz treatment group was sprayed with 10 mL of amitraz diluted in acetone (i.e., 4.3 mg of amitraz / 100 mL of acetone) applied to both sides of the frame using a separate 750 mL all-purpose sprayer. The amount used was chosen based on the high concentration of amitraz detected in wax samples collected by [15] (Table 1).

**Table 1:** Summary of pesticide detections in wax samples from commercial beekeeping operations across the United States^a^.

Pesticide	Class	Detection rate (%)	High detection (ppb)
Fluvalinate	Pyrethroid	98.1	204000
Coumaphos	Organophosphate	98.1	91900
Chlorpyrifos	Organophosphate	63.2	890
DMPF (amitraz)	Formamidine	60.5	43000
Chlorothalonil	Fungicide	49.2	53700

^a^ Table adapted from Mullin et al. 2010 [13], Table 4

In 2017, eight drone foundation frames were allocated to one of two treatment groups each, and sixteen were allocated to the untreated control group. Each frame allocated to the first treatment group was coated with 10 mL of a miticide solution containing fluvalinate and coumaphos, the two most ubiquitous beekeeper-applied miticide products found in wax by [15]. We diluted 20.4 mg of fluvalinate and 9.2 mg of coumaphos in 100 mL of acetone and applied the mixture to both sides of the frame using a separate 750 mL all-purpose sprayer. Each frame allocated to the second treatment group was then coated with 10 mL of a pesticide solution containing the fungicide chlorothalonil and the insecticide chlorpyrifos, the two most ubiquitous field-applied agrochemicals found in wax [15]. We diluted 5.4 mg of chlorothalonil and 0.09 mg of chlorpyrifos in 100 mL of acetone and applied the mixture to both sides of the frame using a separate 750 mL all-purpose sprayer. All pesticides were purchased from Sigma-Aldrich (St. Louis, MO, USA). In all treatment groups, the acetone was allowed to evaporate and the frames were not used until at least one day after spraying. In 2016, frames were paired off such that two control frames accompanying two treatment frames in each hive, resulting in the use of four hives. In 2017, two control and two treatment frames were allocated to each of four hives per treatment group for a total of eight hives, similar to the previous year.

### Drone rearing and capture

**Fig 1:**
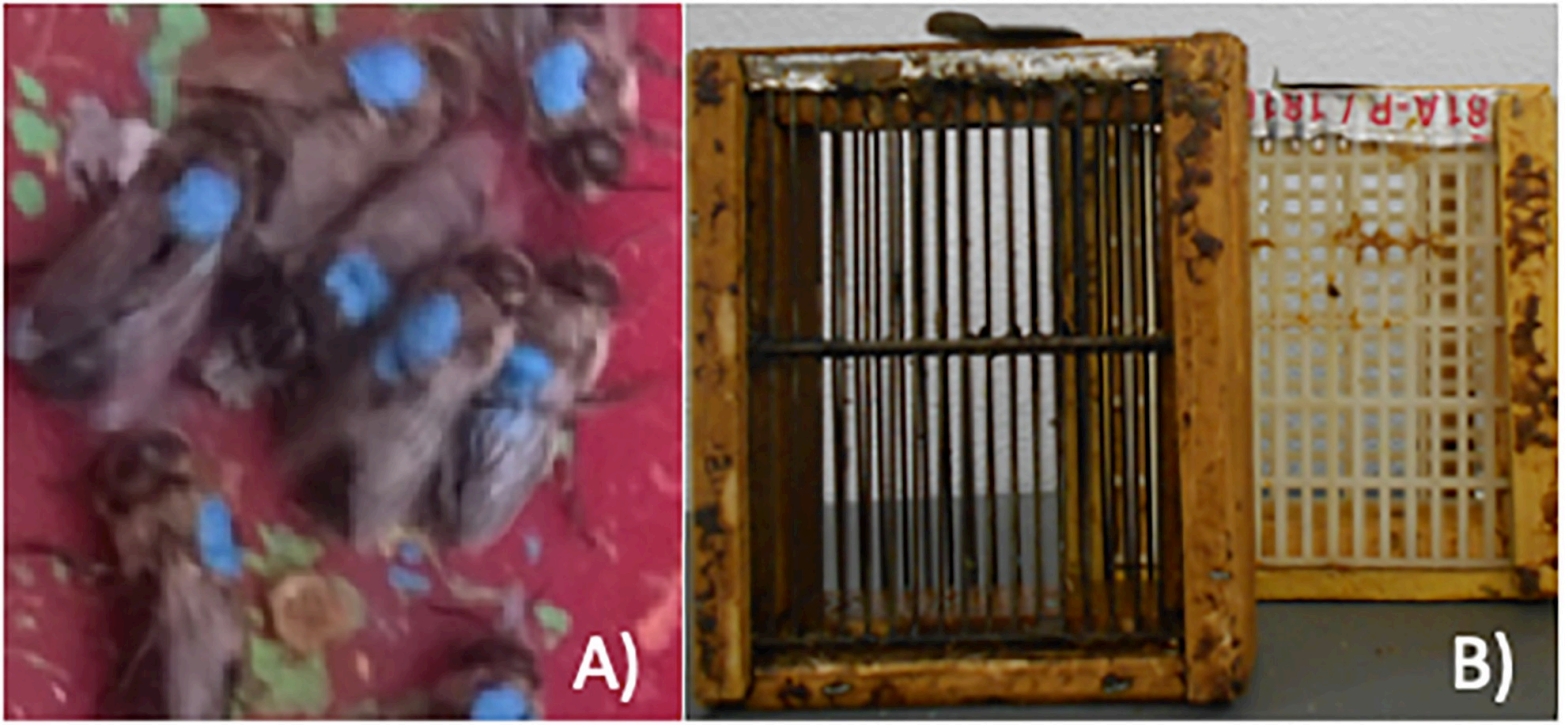
Drone labeling and collection in holding cages. A) Drones collected one day post emergence were marked with paint on the mesonotum before being returned to their host hive. Using different colors of paint helped to distinguish experimental drones from regular drones and to track their age. B) Drone cages were used to increase the recapture rate of amitraz-treated and control drones in 2016.

Experimental frames were placed into host colonies each year, allowing the workers to build comb and the queen to lay drone-destined eggs at will. Experimental frames with brood remained in their respective host hives throughout larval development. Following pupation and the capping of cells, experimental frames were removed from their host hives and placed individually into 5-frame nucleus hives (“nucs”) approximately one day prior to the anticipated emergence of adults. We did so by opening a few cells to check if the pupae were fully sclerotized and had a dark exoskeleton. One frame containing honey and pollen and a small group of young workers was included in each nuc box to tend to new drones as they emerged. The nucs were placed in an incubator at 34°C and ≈75% relative humidity. Emergence of adults was monitored daily, and emerged drones were gathered and marked on the mesonotum using latex paint (Fig 1A). A different color was used to label drones in each treatment group and for each day of drone emergence to keep track of their age.

In 2016 all marked drones from each frame were placed in a drone cage (16.51 cm x 15.24 cm x 7.62 cm) consisting of a wooden frame and two sliding queen excluder side panels (Fig 1B). Once loaded with drones, the cage was placed back in the original host hive. The use of drone cages was implemented to increase the recapture rate, which was low in preliminary studies when drones were allowed to roam freely outside the hive. Caged drones were retrieved at 10 and 18 days post emergence, representing sexually immature and a sexually mature subsets of drones, respectively [4].

In 2017 we did not use the confinement cages were not, and instead, we placed a queen excluder between the hive’s brood box and the bottom board, allowing drones to roam freely within the hive, but preventing them from going outside. This change was implemented due to a lack of success in obtaining drones that survived in the cages. Drones were captured only at 10 days post emergence in 2017, given that we were unable to find marked drones at 18 days post emergence.

### Semen collection and sample preparation

Drones captured 18 days post emergence in 2016 underwent forced ejaculation through the application of pressure on the thorax and abdomen, which triggers the eversion of the endophallus [9]. Semen samples of approximately 1μL in volume were collected using a 10 μL pipette and were transferred individually to micro centrifuge tubes containing 100 μL of saline solution (0.24 g HEPES, 0.88 g NaCl and 1 g BSA diluted in 100 mL of deionized water) to extend sperm longevity for viability analysis [27,28].

In both years, semen collection from drones captured 10 days post emergence was done by decapitating the drones and immediately dissecting their reproductive tract. The seminal vesicles were removed from the rest of the tract and crushed into an micro centrifuge tube containing 100 μL of saline solution, allowing the sperm and seminal fluid to mix with the solution. Each semen sample was then combined with 3 μL of sybr-14 (Life Technologies, Carlsbad, CA, USA) solution, which was derived from a dilution of 4 μL of syber-14 in 196 μL of 0.1 M phosphate buffered saline (PBS), and 3 μL of propidium iodide solution (Life Technologies, Carlsbad, CA, USA), which was derived from diluting 50 μL of propidium iodide in 50 μL of 2x PBS. These solutions differentially stained viable and non-viable sperm, respectively, as shown previously [29]. Prepared samples were then gently shaken and incubated in a dark cabinet at room temperature for eight minutes before being used for viability analysis.

### Sperm viability analysis

Sperm viability was analyzed using a Nexcelom Cellometer Vision CBA Image Cytometer (Nexcelom Biosciences LLC., Lawrence, MA) at the Heep Center of Texas A&M University, College Station, TX. A 20 μL aliquot of each prepared semen sample was loaded into a cell counting chamber of a cartridge for the Nexcelom Cellometer Image Cytometer and allowed to rest for five minutes prior to processing. The cell counter then automatically quantified the number of viable and non-viable sperm cells in each sample, from which a sperm viability estimate was then estimated.

### Statistical analysis

We tested for differences in sperm viability between treatment and control groups in 2016 and 2017 using Student’s *t*-tests using using the JMP v12 statistical software (SAS Institute Inc., Cary, NC). We set the level of statistical significance for all tests at α = 0.05. All descriptive statistics are reported as the mean ± the standard error of the mean (S.E.M.).

## Results

**Fig 2:**
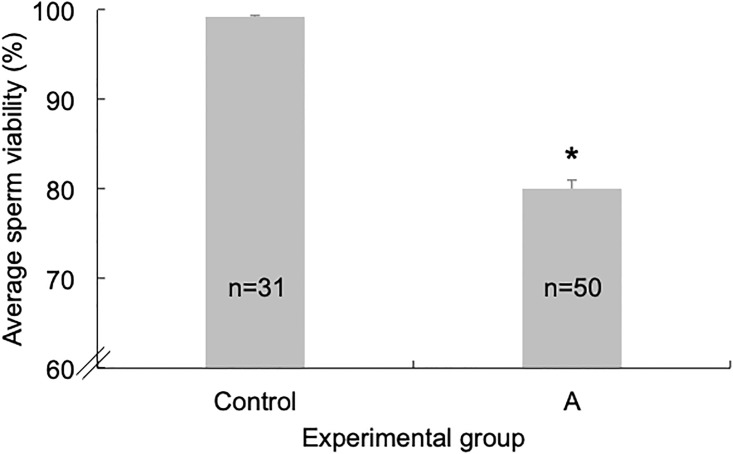
Sperm viability in drones exposed to amitraz during development. Average (± S.E.M.) sperm viability observed in 2016 for drones (10 and 18 days old) reared on frames coated with pesticide-free wax (Control), or wax contaminated with the miticide Amitraz (“A” experimental group). The asterisk above the columns represent a value of *P*<0.05.

We collected sperm viability data from 31 drones in the control group and 50 drones in the amitraz-treated group in 2016. Although four hives were used for drone rearing that year, only one hive successfully reared drones on both experimental and control frames. Overall, the presence of amitraz in the wax that coated the experimental frames used to rear drones resulted in an overall decrease in drone sperm viability. Given that we found no significant difference in sperm viability between drones collected at 10 days and 18 days post emergence in either the pesticide-free control group (*t* = 0.06, *P* = 0.48), or the amitraz-treated group (*t* = 0.23, *P* = 0.83), we pooled the data from both age cohorts and performed an overall comparison between treatment groups using all the drones reared in each treatment group. The average sperm viability of drones in the amitraz-treated group was 80.1% ± 1.0%, which was significantly lower than the viability of 99.2% ± 0.2% of drones in the control group (*t* = 19.8, *P*<0.0001; Fig 2).

**Fig 3:**
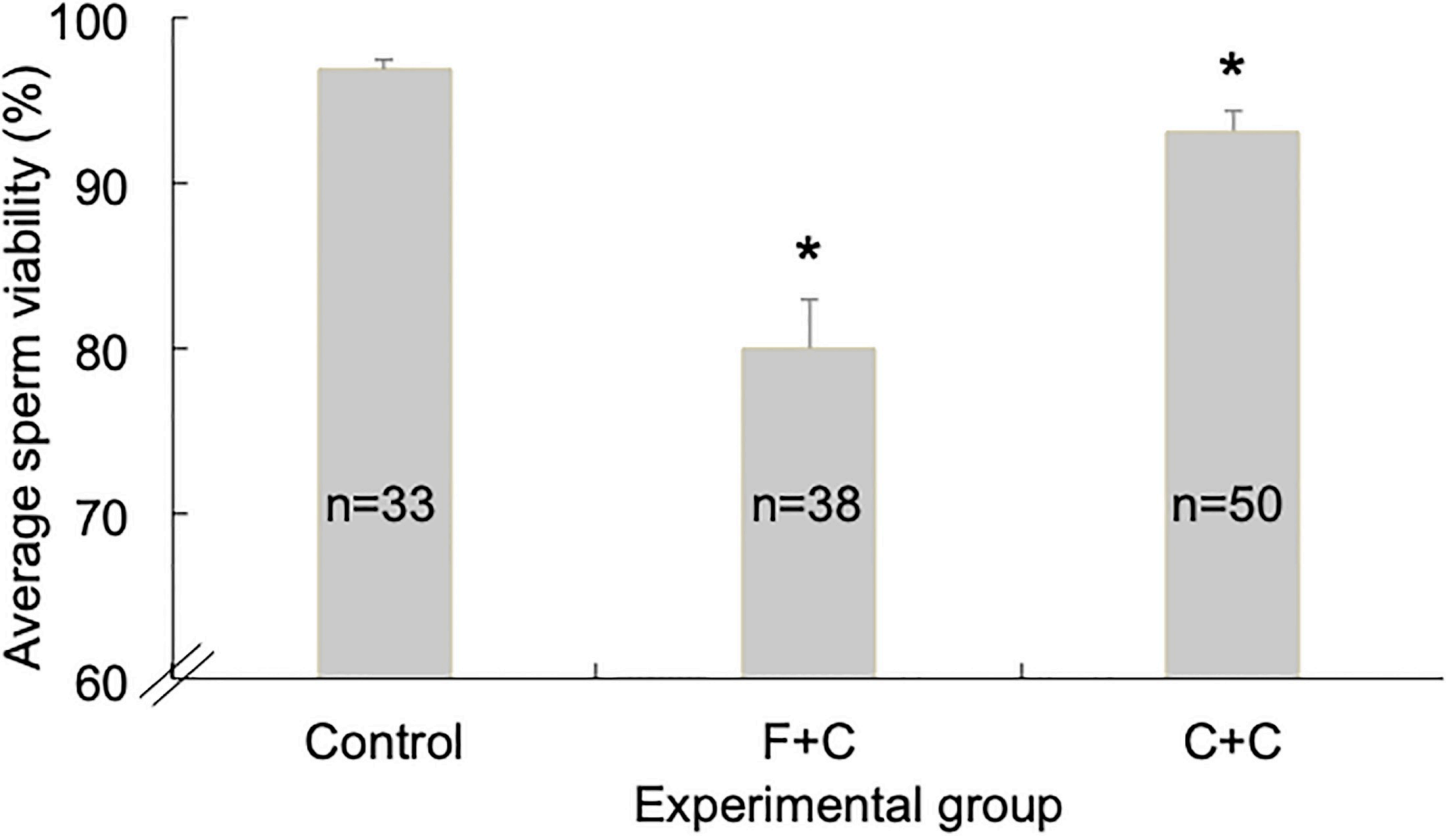
Sperm viability in drones exposed to miticides and agro-chemicals during development. Average (± S.E.M.) sperm viability observed in drones (10 days old) reared in 2017 on frames coated with pesticide-free wax (Control), wax contaminated with a mixture of the miticides Fluvalinate and Coumaphos (“F+C” experimental group), and wax contaminated with a mixture of the agrochemicals Chlorothalonil and Chlorpyrifos (“C+C” experimental group). The asterisk above the columns represent values for which *P*<0.05.

Likewise in 2017, we found that drones reared in frames coated with wax exposed to miticides or agro-chemicals had lower sperm viability than those reared in pesticide-free frames (F = 22.5, *P*<0.0001). We sampled sperm viability from 33 drones in the control group, 38 drones in the miticide treatment group, and 50 drones in the agro-chemical treatment group. Drones in the miticide treatment group, which consisted of a mixture of fluvalinate and coumaphos (“F+C” treatment), had an average sperm viability of 80.0% ± 2.87%, which was significantly lower than the viability of 96.9% ± 0.64% in the control group (*t* = 5.73, *P*<0.0001; Fig 3). Drones exposed to the agrochemical treatment group, which consisted of a mixture of chlorothalonil and chlorpyrifos (“C+C” treatment), had an average sperm viability of 93.2% ± 1.16%. While sperm viability of drones in the C+C group was higher than the viability of drones in the F+C group, it was nevertheless significantly lower than the sperm viability of drones in the control group (*t* = 2.43, *P* = 0.009; Fig 3), despite having a small numerical disparity from the control group (3.7%).

## Discussion

This is, to our knowledge, the first study looking at the effects of pesticide exposure of the brood-rearing wax environment on drone sperm viability. We found that the beekeeper-applied miticides amitraz, fluvalinate and coumaphos, and to a lesser extent, the field-applied agro-chemicals chlorothalonil and chlorpyrifos, impair drone reproductive quality. In particular, when applied at field-relevant concentrations, we found a combinatorial effect of fluvalinate and coumaphos, as well as a combinatorial effect of chlorothalonil and chlorpyrifos, on drone sperm viability. Our results complement previous studies of the sub-lethal effects of commonly used miticides and agro-chemicals on drone reproductive health [19–21,].

Pesticide residue analyses were not conducted to verify chemical concentrations in the wax combs used to rear drones. In addition, the initial coat of wax applied to an experimental frame was not precisely measured prior to the drawing out of comb on the pesticide-treated foundation. Thus, drone larvae were likely exposed to pesticide concentrations lower than those initially applied to the experimental frames. While pesticide diffusion rates in beeswax have not been assessed to date for the pesticides we used, Wu et al. (2011) noted substantial accumulation of pesticide residues within three months, including fluvalinate and coumaphos, in wax that was previously free of contamination [30]. In addition to their apparent rapid diffusion, fluvalinate and coumaphos undergo slower degradation in beeswax with an approximate half-life of five years [31].

We did not find differences in sperm viability between drones collected at 10 or 18 days post emergence, suggesting that the effects of exposure to pesticides occurs early in a drone’s developmental process, which is the key period when spermatogenesis occurs [5,22,23]. An interesting aspect of drone biology is the process of sexual maturation, which describes the migration of the sperm generated during development through the reproductive tract [32,33]. Sperm cells migrate from the testes to the seminal vesicles where they receive nourishment and support from the mucus glands, the accessory reproductive organs that provide a mucous secretion during ejaculation to maintain sperm viability [32,33]. This process directly precedes the completion of maturation, when semen is transferred to the drone’s endophallus. Various estimates have been proposed for the time needed to complete sexual maturation in drones, from low estimates of 6 to 8 days [34,35] to high estimates of two weeks or more [36–38]. In preliminary experiments we successfully obtained semen samples through forced eversion of the endophallus 18 days after emergence, which is a longer post emergence period than the average 10 to 12 days that is usually reported in the literature [4]. Interestingly, we were unable to obtain semen from drones younger than 18 days post emergence in both years. We lost dozens of samples that could have yielded sperm viability results but that were sampled too early (at about 10 to 14 days post emergence), which is a timeline at the upper end of what has been reported for sexual maturity in drones [36–38]. As a result, we were forced to obtain semen through dissecting drones and accessing the seminal vesicles at 10 days post emergence. With this procedure we averted the low survival rate among experimental drones that occurred when they were allowed to complete sexual maturation more than 10 days after emergence.

Our poor success in obtaining experimental drones at 18 days post emergence suggests the possibility that the confinement techniques that we used to keep the drones from going outside the hive may have affected their maturation and longevity. One study recently showed that drone sperm undergoes a high degree of environmental pressure and is highly sensitive to fluctuations in temperature and threats to the immune system [1]. However, that study was conducted mainly in laboratory conditions, and it has been shown that rearing drones in the laboratory produces drones with reproductive traits that are different from those of drones reared in field conditions [39]. Another possibility is that pesticide exposure during development not only causes lower sperm viability in sexually mature drones, but also lowers their lifespan, which is another way in which their reproductive fitness might be compromised by pesticide exposure. The effect of environmental factors including exposure to pesticides, nutrition, and temperature on drone fertility remain poorly understood and should be further tested during drone development in field conditions rather than in laboratory settings.

The ubiquitous presence of pesticide residues inside hives not only affects drone development and reproductive health, but it may also indirectly affect the reproductive quality of queens that mate with pesticide-exposed drones. Queen longevity is a function of her brood production rate, with reductions in productivity often triggering supersedure [40]. Because drones are the source of half of the genetic material required for the production of female workers, a queen that mates with substandard drones may run out of viable sperm sooner than normal. Interestingly, the long-held view that queens retained only viable sperm after mating [41] has been challenged by recent evidence of the retention of non-viable sperm in the queen’s spermatheca [41,42]. The negative impact of common in-hive pesticides on drone fertility may confer queens with a sizeable proportion of non-viable sperm from their mates, which may contribute to a reduction in their supply of viable sperm needed to fertilize eggs [43]. This apparent consequence was demonstrated in queens artificially inseminated with semen from drones reared on syrup tainted with the insecticide fipronil [11]. Sperm stored in the spermathecae of these queens had significantly lower viability with respect to queens inseminated with semen from control drones [11]. This occurrence of compromised sperm stored in queen spermathecae was also observed in queens reared on pollen contaminated with the neonicotinoids thiamethoxam and clothianidin [44], and exposure to imidacloprid resulted in a similar outcome on spermathecal sperm stores at varying doses [45]. Furthermore, Baer (2005) suggested that drone ejaculate size directly affects successful sperm storage in queens following mating, while successful fertilization depends on the quality of individual sperm cells [5].

Indeed, understanding the factors that affect sperm quality in drones will help us better address the issues of drone and queen fitness. In this regard, beekeepers seem to be inadvertently reducing drone fitness through the use of miticides to combat *Varroa* mites. Honey bee exposure to agro-chemicals presents a complicated and perhaps unavoidable risk, given the near ubiquitous presence of such pesticides in cultivated and uncultivated areas [46,47]. The widespread breadth of contamination of floral resources may also lead to a colony’s continuous exposure through pollen and other food resources. In light of our findings, it is advisable for beekeepers to reduce the influence of exposure to miticides on drone fertility by formulating best management practices that include systematically replacing old frames with new ones, allowing the bees to produce new (cleaner) wax, lowering the exposure of colonies to field-applied pesticides, and reducing the use of in-hive miticides, all of which will likely help them improve colony health and productivity.

## Supporting information

S1 Data Raw sperm viability data from drones reared in 2016 and 2017. Drones in 2016 were reared on frames coated with pesticide-free wax (Control), or wax contaminated with the miticide Amitraz (“A” experimental group). Drones in 2017 were reared on frames coated with pesticide-free wax (Control), wax contaminated with a mixture of the miticides Fluvalinate and Coumaphos (“F+C” experimental group), and wax contaminated with a mixture of the agrochemicals Chlorothalonil and Chlorpyrifos (“C+C” experimental group).
(XLSX)
